# Grievance-fueled sexual violence

**DOI:** 10.3389/fpsyg.2023.1070484

**Published:** 2023-03-14

**Authors:** Tamsin Higgs, Rajan Darjee, Michael R. Davis, Adam J. Carter

**Affiliations:** ^1^Department of Psychology, The University of Montreal, Montreal, QC, Canada; ^2^International Centre for Comparative Criminology, Montreal, QC, Canada; ^3^Institut National de Psychiatrie Légale Philippe-Pinel, Montreal, QC, Canada; ^4^Tasmanian Health Service (THS), Hobart, TAS, Australia; ^5^School of Health Science, Centre for Forensic Behavioural Science, Swinburne University of Technology, Alphington, VIC, Australia; ^6^Department of Psychiatry, Monash University, Melbourne, VIC, Australia; ^7^Department of Psychiatry, The University of Melbourne, Parkville, VIC, Australia; ^8^HM Prison and Probation Service,London, United Kingdom

**Keywords:** sexual offending, violence, grievance, agonistic continuum, interventions

## Abstract

The grievance fueled violence paradigm encompasses various forms of targeted violence but has not yet been extended to the theoretical discussion of sexual violence. In this article, we argue that a wide range of sexual offenses can be usefully conceptualized as forms of grievance fueled violence. Indeed, our assertion that sexual violence is often grievance fueled is unoriginal. More than 40 years of sexual offending research has discussed the pseudosexual nature of much sexual offending, and themes of anger, power, and control – themes that draw clear parallels to the grievance fueled violence paradigm. Therefore, we consider the opportunities for theoretical and practical advancement through the merging of ideas and concepts from the two fields. We examine the scope of grievance in the context of understanding sexual violence, and we look to the role of grievance in the trajectory toward both sexual and nonsexual violence, as well as factors that might distinguish grievance fueled sexual from nonsexual violence. Finally, we discuss future research directions and make recommendations for clinical practice. Specifically, we suggest that grievance represents a promising treatment target where risk is identified for both sexual and nonsexual violence.

## Introduction

Grievance fueled violence (GFV) is a paradigm referring to violent threat from lone individuals with complex grievances, that has come to include various forms of targeted violence, including organized terrorism, lone actor terrorism, mass murder, school shooting, educational and workplace violence, stalking, public figure harassment and assassination, familial and intimate partner homicide, cyberthreats, insider threats, and hate crimes (Meloy and Hoffmann, 2021). There is a growing body of academic work on theory and research to inform our understanding of these behaviors. Threat assessment and management, the practical approach to identifying and analyzing threats of such violence, and then intervening to prevent it, has gone from an emerging field to a burgeoning area of interdisciplinary practice (Meloy and Hoffmann, 2021). As the editors of this special edition have pointed out, the unifying concept of GFV provides a potential framework for co-constructed knowledge between fields that have largely developed separately from each other in terms of theory, research, and practice.

Consequently, it is interesting to note that this expanding field has rarely, if ever, included sexual violence within its scope. The seminal work in the field, now in its second and deservedly award-winning edition, the *International Handbook of Threat Assessment*, has less than a dozen of its over seven hundred pages mentioning sexual violence, most of them related to false claims of rape and none focused clearly on managing threats of sexual violence (Meloy and Hoffmann, 2021). So, one might assume that the GFV paradigm and its pragmatic branch, threat assessment and management, are irrelevant to sexual violence and vice versa. So much has been written about risk assessment and management with those who commit sexual offenses (Davis and Ogloff, 2008; Darjee and Russell, 2012; Russell and Darjee, 2013; Craig and Rettenberger, 2016; Kemshall, 2017; Helmus, 2018; Levenson, 2018; Brankley et al., 2021; Raymond et al., 2021), and although threat and risk assessment and management overlap (Meloy et al., 2021), and some behaviors manage to sit across both camps (e.g., stalking and intimate partner violence), many behaviors seem to fall in one or other, and sexual offending practice is squarely in the risk one. Traditionally with sexual offending the emphasis has been on preventing recurrence after an offense has occurred, rather than preventing an offense in someone with concerning thoughts or behavior. Although there has been a growing focus on primary and secondary prevention of sexual violence (Knack et al., 2019; Assini-Meytin et al., 2020), this has largely been focused on child sexual abuse, and the threat assessment paradigm has not yet been invoked in the sexual offending field.

Given this potential shift toward considering the commonalities among apparently different behaviors within the GFV paradigm, should sexual violence, or at least some forms and aspects of sexual violence, come within its scope? And what can theory, research, and practice with those who commit sexual violence contribute to the expanding GFV field? In this paper we will consider these two questions and argue that: At least some, if not most, sexual violence is grievance fueled and targeted; so called “vindictive rape” is the archetype of grievance fueled sexual violence, but other aspects of sexual violence also invoke concepts of grievance; there are obvious overlaps between non-sexual GFV and sexual violence, especially forms fueled by the grievances of males toward females; and, grievance is a relevant concept in sexual violence even when it is not committed by a male toward a female. We then consider the theoretical, research and practice implications of bringing sexual violence, or certain forms of it, within the GFV paradigm.

## Grievance fueled violence

The term “grievance” can be used in three ways. A person may suffer a grievance, they may be in the state of having a grievance, and/or they may take out a grievance. So firstly, it may refer to the event or circumstance that provokes a person to feel aggrieved. This may be an actual or perceived wrongdoing or unfairness, and either distal or proximal in time. Secondly, it refers to the psychological state of being or feeling aggrieved with interacting perceptual, cognitive, emotional, and behavioral components. A person may not necessarily have suffered an actual grievance to feel aggrieved, although they will usually at least perceive themselves to have. Thirdly, grievance relates to the actions taken, and remedies sought, to communicate and find a resolution that satisfies the aggrieved person, which may include threats and violence. Many, perhaps most, people with a grievance do not resort to an external process in an attempt to resolve it, but some do take out or enact a grievance. As will be set out below, all three of these concepts of grievance are relevant to sexual violence.

The term “fueled” can be used in two ways: to supply or power so as to start, put into motion or perpetuate; and to cause to intensify or escalate. Again, both concepts have relevance to the way grievances can lead to sexual violence, which may repeat or escalate through this fueling. A concept that is central to targeted violence is “fixation,” an obsessive interest in or feeling about someone or something. This emerges from the state of feeling aggrieved and encompasses ideation, fantasy and behavior which is ruminative, preoccupying and self-perpetuating, but which may wax and wane depending on psychological state (e.g., depression or anger), environmental triggers (e.g., seeing the target or being reminded of the target) and social context (e.g., isolation, lack of occupation, rejection). And again, aspects of fixation are relevant to sexual violence, potentially interacting with the added dimensions of sexual fantasy, sexual arousal, and sexual preoccupation, which are known to “fuel” sexual violence. The goal → intent → behavior sequence has been articulated as a key underpinning of threat assessment and management (Meloy et al., 2021), and the concept of “pathway” (Meloy, 2018) has been employed to refer to the process and indicators that may signify a person is progressing from being predisposed to acting on their grievance. In a similar vein, pathways to sexual violence, including distal and proximal factors, and the progression from psychosocial predisposition to offending, including consideration of environmental and victim factors, have been proposed and researched (Hudson et al., 1999; Proulx et al., 2014; Stefanska et al., 2015; Brouillette-Alarie and Proulx, 2019; James and Proulx, 2020).

Before considering the application of concepts from the GFV paradigm to sexual violence, it is relevant to note examples where problems with sexual behavior and overt sexual violence have been associated with “classic” types of GFV (i.e., those listed at the start of this paper). Table 1 shows some such examples, these being either well known cases in the public domain, or from the authors’ practice. All these cases involved sexual violation and behaviors which would be sexual offenses in most jurisdictions. The term grievance fueled sexual violence could be applied across these cases, and some show evidence of sexual violence both within and outside of the commission of GFV. But the role of sexual issues (such as deviance, attitudes, arousal, attraction, intimacy, preoccupation) in interaction with non-sexual factors which underpin GFV (including all three types of grievance) is clearly diverse; the motivation for and the role of the sexual violence in each case is quite different; and the place of the sexual violence in the temporal sequence of behaviors clearly varies.

**Table 1:** Examples from the authors’ practice or the public domain of grievance fueled violence involving problems with sexual behavior.

Lone actor or group	Description of the offense
Individual male	Mass school shooting after being suspected of committing child sexual abuse, being stopped from working with children’s organizations, which was apparently one of the grievances fueling the shooting (Cullen, 1996)
	Stabbed wife to death in a jealous rage then had sex with her dead body (Meloy, 1996)
	Repeatedly raped his partner as part of a pattern of coercive control, then strangled her to death when she said she was leaving (Chantler et al., 2020)
	Took several female school students hostage, sexually assaulted them and then shot and killed one before turning the gun on himself (Dishman et al., 2011)
	Stalked a film director, was caught at the director’s house several times, was sexually obsessed with the director, and articulated a plan to handcuff and rape him (Meloy and Mohandie, 2008)
	Having convictions for rape and murder, was infatuated with a male pop star, was offended at not getting a reply to his fan mail, and then plotted from prison with another man to have him castrated and killed, as well as castrating two other males unrelated to the pop star (Pearse, 2012)
	Stalked a female celebrity, at one point sending her pornography and sex toys (Hoffmann and Meloy, 2008)
	Disgruntled at being “dumped” by his girlfriend, posted explicit sexual images of her online and circulated them to her friends and family
	With a grievance toward a politician, wrote letters to the politician in which he threatened to rape his daughter and put sexually demeaning posts online about her, and claiming the politician had sexually abused her
	After a short period of dating a co-student, stalked her after being rejected, and then broke into her room and raped her
	Thought to have committed previous rapes and a sexual homicide, but with insufficient evidence to charge or convict; abducted, raped and attempted to kill a woman who ended a relationship with him and tried to kill her child; then while in prison he married another woman, who left him and fled far away when he was on parole; during a subsequent period in prison was found to have a diary expressing violent sexual fantasies and getting revenge, then in the community managed to locate her and was arrested and convicted for conspiracy to murder, having accumulated materials indicating his intent to rape and kill her
Individual female	Stalked a psychiatrist, tried to force herself sexually on him, took a used condom from his waste bin, put his semen in her vagina and on her underwear, and then made a false accusation of rape (Lewis, 2006)
Group	Terrorists killed all of their hostages, mutilating the genitals of one (Large, 2012)
	Klu Klux Klan members abducted, tortured, castrated with a razor blade, and burnt to death an African American man with an intellectual disability (Carter, 2012)

It is important to note that different types of problematic and offending behaviors, including sexual ones, may be manifested by one individual either at the same time or separately. This is in keeping with research findings that show that: People who commit sexual offenses, particularly those with adult victims, are not “sexual offending specialists” (Lussier et al., 2008; Howard et al., 2014); when a sexual offender recidivates he is more likely to commit a non-sexual than sexual crime (Hanson and Morton-Bourgon, 2005); and people presenting with one type of problematic behavior (stalking, threats, sexual offending, arson or violence) often demonstrate a second one (McEwan and Darjee, 2021). This points to potentially common factors, which may be biological, psychological, social, cultural and/or environmental, underpinning different problematic behaviors. Therefore, there is potential for more holistic and less siloed approaches to intervention and prevention, straddling, or perhaps making defunct, the apparent boundaries between them.

## Grievance fueled sexual violence

While the GFV literature has largely neglected the area of sexual violence, grievance and hostility are well established risk factors for sexual recidivism. Indeed, a meta-analysis by Mann et al. (2010) described this as an *empirically supported risk factor*. Based on 11 studies, a relatively small but reliable effect was found (average *d* = 0.20, 95% CI [0.09, 0.31], *Q* = 13.58, *p* > .05). Mann and colleagues stated that this risk factor “involves the perception of having been done wrong by the world, feeling that others are responsible for their problems, and wanting to punish others as a consequence. Offenders with this schema are preoccupied with obtaining the respect they desire from others and frequently ruminate on vengeance themes. They have difficulty seeing other people’s point of view and anticipate further wrongs will be perpetrated against them” (pp. 202–203).

While there are a range of typological classifications for rapists, almost all can be viewed as variants of the one initially described by Cohen et al. (1969; see also Groth et al., 1977; Hazelwood, 1987; Canter and Heritage, 1990). The most well-validated of these variants is the Massachusetts Treatment Center Rapist Typology, currently in its fourth iteration (MTC:R4; Knight, 2010; Knight and Sims-Knight, 2016; see also Prentky et al., 1985; Knight and Prentky, 1987; Knight and Prentky, 1990; Prentky and Knight, 1991; Knight, 1999; Knight and Guay, 2006). There are essentially six primary types of rapist in the MTC system.^1^ Three of these involve largely instrumental aggression: The *opportunistic* (based on impulsive exploitation), the *non-sadistic sexualization* (based on feelings of social and sexual inadequacy), and the *muted sadistic* (involving symbolic forms of sadism but no gratuitous violence^2^). The remaining three are characterized by high levels of expressive physical violence: the *overt sadistic*, the *pervasively angry*, and the *vindictive*. The latter was new in the MTC:R3 system. It was initially envisaged as a misogynistic variant of the pervasively angry type, the key difference being that the pervasively angry rapist was characterized by global and undifferentiated anger while the vindictive type’s anger was exclusively misogynistic and directed toward women. In both cases the offender is essentially punishing the victim using their penis and their fists and the level of expressive aggression is far beyond that necessary to simply force the victim to comply. Indeed, the physical punishment will routinely continue after the sexual assault has ended and may lead to the victim’s death.

It is clear that both pervasively angry and vindictive rapists, along with arguably at least some of the overtly sadistic, reflect motivations for offending that could be viewed as grievance fueled. Whether the grievance is global (pervasively angry) or misogynistic (vindictive), the offender is seeking to punish a victim for actual or perceived wrongs that they have suffered. This is perhaps most clear when one views the behavior of men who have raped through the lens of the *victim role model* (Canter, 1994; Canter and Youngs, 2009, 2012). This model posits that empathy deficits lead to all violent and sexual offenders assigning their victims to a role of *person*, *object*, or *vehicle*. As the names suggest, the victim as vehicle role is one in which the victim is seen as a vehicle or means through which the offender’s anger or other emotions can be expressed. The pervasively angry, vindictive, and overtly sadistic rapists of the MTC typology can be viewed in this way. At a more general level, all three of the victim roles can be envisaged as a continuum of grievance, starting as a grievance toward themselves (victim as person; non-sadistic sexualization and muted sadist types), a selfish grievance against the world where one can take what they want (victim as object; opportunistic type), or a decidedly more clear grievance against either females or the wider world (victim as vehicle; overt sadistic, pervasively angry, and vindictive). However, it is only the latter, at the end of this hypothesized continuum, where the offender chooses to severely punish their victim for the grievances they feel toward females or the wider world. As conceptualized by Hanson (2020), vindictive rape is “considered a form of retributive justice in which the perpetrator punishes the female victim for perceived transgressions against sexual norms” (p. 1).

### Sexual sadism, other paraphilic disorders and grievance fueled sexual violence

While the above discussion suggests grievance can be envisaged as underpinning, at least to some degree, practically all sexual victimization of adult females, it is clearly not the only contributing factor. One of the factors that would arguably appear to be external to grievance is that of paraphilic sexual interests and disorders. It seems clear that those with overt behavioral manifestations of sexual sadism disorder share a seemingly large amount of grievance with the pervasively angry and vindictive rapist types. However, they are also very much motivated by ritualistic sexual fantasies where the degradation, humiliation, and suffering of their victims is sexually arousing. There has been empirical and theoretical exploration of the developmental antecedents of sexually sadistic behaviors, which indicate that many sexually sadistic offenders have unstable experiences in childhood and adolescence, they are socially isolated and adopt maladaptive coping behaviors and remain socially disconnected in adulthood both in terms of peer and intimate relationships (Higgs et al., 2015, 2017b; Longpré et al., 2018). This provides evidence for the proposition that some sexually sadistic offenders may harbor considerable grievance toward the world or females in particular. However, there are also individuals with powerful sexually sadistic interests and fantasies that never act upon them (Crepault and Couture, 1980; Gray et al., 2003; Dietz, 2014). It is likely that such individuals, whether they find the sadistic fantasies and urges ego-dystonic or ego-syntonic, have a reasonably intact capacity to empathize. Indeed, Dietz et al. (1990) posited that severe sexual sadists “differ from less destructive sexual sadists not in the “severity” of the paraphilia, but in the character pathology that permits them such uninhibited expression of their sexual desires” (p. 173). In addition, it can be hypothesized that those with sexually sadistic interests that they never act upon do not have the underlying grievance that characterizes the overt criminal sexual sadist or vindictive or pervasively angry rapists. This hypothesis also potentially applies to practitioners of BDSM (bondage-discipline, dominance-submission, sadism, masochism) that is not directed at non-consenting victims and is not therefore forensically relevant: BDSM practitioners are typically well socially adapted and have healthy personality profiles, have stable intimate relationships, and are not sensitive to rejection (Connoly, 2006; Wismeijer and Van Assen, 2013; Landgraf et al., 2018; De Neef et al., 2019; Brown et al., 2020). In other words, BDSM that is not forensically relevant is not grievance-fueled. Although sexual pleasure is derived from apparently extreme acts of intimacy, any coercion in fantasy or reality is strictly consensual.

**Figure 1:**
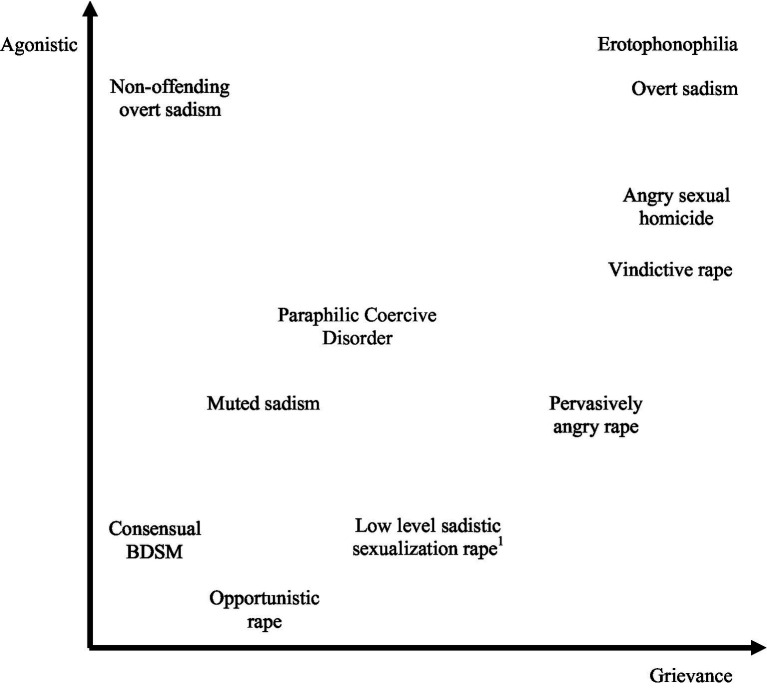
The grievance-agonistic model of grievance fueled sexual violence. ^1^Non-sadistic sexualization rapist in the MTC typology.

More recent explorations by Knight et al. (2013) and Longpré et al. (2020) have led to the proposal of an *agonistic continuum*. They cogently argue that the proposed for and rejected from DSM-5 diagnosis *Paraphilic Coercive Disorder* is essentially a form of sadism that occurs earlier along the same coercive continuum. It is our opinion that this agonistic continuum can actually be expanded to encompass a range of sexual behaviors from consensual BDSM activity to overt sexual sadism and erotophonophilia (paraphilic sexual homicide). While this could also be argued to be a continuum of grievance, we propose that the expanded agonistic continuum exists alongside a separate grievance continuum that is informed by the victim role model. Consequently, we propose a conceptualization of grievance fueled sexual violence as the intersection of two axes: Grievance and the agonistic continuum (Figure 1). While the orthogonal representation of these two axes in our model reflects our current theorization (empirically informed but as yet untested), in the case of an individual who could be positioned on the lower extremity of the two dimensions, the relationship between sexual drive and violence is at its most indirect. For example, the *power rapist* described by Groth and Birnbaum (1979), who uses force to gain victim compliance but holds cognitive distortions around the victim’s receptivity and potential for eventual reciprocal arousal. A functional analysis of the offending behavior using the Grievance-Agonistic model would consider the interplay between the grievance axis, which might be characterized in a case like this by ambivalent attitudes toward women; hostility combined with unrealistically positive views of women and desire for intimacy (Schippers and Smid, 2021), and sexual arousal experienced in relation to coercion and whether the individual gained any positive emotional reward from hurting or humiliating the victim, on the sadism axis.

With increasing intensity on the sadism axis, the *vindictive rapist* (Knight and Prentky, 1990) harbors anger and resentment toward women and he chooses sexual violence because he considers this the worst way to aggress another person. Knight and Prentky’s (1990) pervasively angry and vindictive types were both initially thought to have little in the way of sexualization and fantasy in their offenses. However, this has since been challenged. Indeed, subsequent studies have found relatively high degrees of sexualization among vindictive rapists, to the point that some vindictive rapists appear more similar to the overtly sadistic type (Proulx and Beauregard, 2014). As such, it has recently been proposed that vindictive rapists themselves exist on a continuum from the predominantly angry to the more sadistic, with the common underlying feature being high levels of misogyny (Davis, 2022). For the predominately angry, aggression has not become eroticized, but despite not having developed sadistic sexual fantasies, this individual seeks to degrade and humiliate his victim (Knight and Prentky, 1990), and he is able to overcome the inhibitory effect of anger in order to use sex as a weapon. Alternatively, he might experience erectile inadequacy, or impotency during the offense; non-penetrative sexual assault serves to reinstate his position of power and express his anger. Thus, on the grievance axis, victims are seen as deserving of punishment; on the agonistic axis, succeeding to dominate and degrade the victim is cognitively and emotionally rewarding.

Finally, on the most extreme end of the agonistic continuum is the severe, or overt sexual sadist. What is worth noting here is that while various typological approaches to understanding sexual violence consistently identify a subtype of offender said to be primarily driven by sexual sadism suggesting that this is a distinct, special type of offender, at the same time, the psychological function of sexually sadistic offending originates in the experience of grievance: Severe sexual sadism is the “sexual transformation of anger and power” (Groth and Birnbaum, 1979, p. 44); and features multiple dimensions, one of which is sadistic sexual fantasies, others include the desire to humiliate and dominate (Longpré et al., 2019). Therefore, our model includes consensual BDSM activity that involves practically no coercion, allowing that a BDSM practitioner may move vertically along the agonistic continuum while remaining low on grievance. Thus, the model asserts that overt sexual sadism expressed in offending behavior depends on the interplay with grievance. Accordingly, it is our contention that grievance and coercive paraphilic sexual interests are distinct, although interacting, and some degree of grievance is arguably necessary for offenses of rape to occur.

## Nonsexual violence underpinned by male grievances toward females

The grievances which fuel some types of sexual violence are also relevant to and shared with non-sexual targeted violence with females as a primary focus. Three specific types of targeted violence will be considered here: intimate partner violence – in which severe physical injuries are more likely to be inflicted by males than females even though intimate partner violence in hetero relationships is often bidirectional (Straus, 2010), noting at the same time that we do not have the scope to discuss violence in LGBTQIA+ relationships; honor-based violence (a term that we will use to avoid ambiguity given its colloquial meaning, while noting the incongruence between reference to honorable versus violent behavior); and violence committed by individuals who identify as involuntarily celibate (incels). All three behaviors share at their core grievances toward women, are considered manifestations of misogynistic and patriarchal sociocultural structures, and alongside rape and sexual assault are described as “gender-based violence” (Division of International Protection, UNHCR, 2021). Here we will compare these forms of violence to sexual violence from a GFV perspective, highlighting commonalties and overlaps, but also considering why individuals go down one or other path with their grievances.

### Intimate partner violence and homicide

Men who commit intimate partner violence, especially those who are coercively controlling (Stark and Hester, 2019), share attitudes, relational difficulties and emotional problems with men who commit rape. Indeed, the most common victims of rape are men’s intimate partners (McOrmond-Plummer et al., 2016b), most if not all coercive control involves sexual abuse (Stark and Hester, 2019), and sexual violence within an intimate relationship is a marker for potential escalation to homicide (Spencer and Stith, 2020). Among men who commit intimate partner violence, attitudes toward women mirror those described in men who commit rape against non-intimate partners: Entitlement; women considered deceitful and untrustworthy; acceptance of use of control or force; women viewed as sex objects or sexually provocative; hostile masculinity; adversarial beliefs about heterosexual relations; women being different and unknowable (Polaschek and Ward, 2002; Blake and Gannon, 2010; Weldon and Gilchrist, 2012; James and Proulx, 2020). The concept of “virtuous violence” can be applied to both intimate partner violence and non-intimate partner rape. This concept is that, from the perpetrator’s perspective, they are morally right to control a victim, expect her to act in certain ways, to take what is theirs by right and to punish her if she infringes this moral code (Fiske and Rai, 2014). Male “proprietariness,” a desire or entitlement regarding exclusive control of women, including concerns about sexual infidelity and strong beliefs about sexual ownership and jealousy, is an overlapping concept which also has relevance to both forms of violence (Wilson and Daly, 1993).

In the intimate partner violence field integrative models of cognitions have attempted to draw together what can appear to be disparate perspectives. Gilchrist (2009) concluded that general antisocial cognition, offense specific cognition, cognition regarding femininity and masculinity, and relationship-specific cognitions each played a role in intimate partner violence. Senkans et al. (2020) proposed the Aggressive Relational Schemas Model of intimate partner violence, incorporating relationship cognitions, antisocial/aggressive cognitions, and gender cognitions. The personality disorders identified as being common in men who commit intimate partner violence and men who commit non-intimate partner rape are very similar (i.e., antisocial, borderline and narcissistic), and the early attachment difficulties which underpin these personality problems and contribute to later emotional and intimacy problems are also similar. The question that arises from this is why do some men target their grievance toward intimate partners, while others target non-intimate partners, and what factors are associated with men who do both? Are they different in terms of the nature and context of the initial grievance, or their state of feeling aggrieved and its development? And how different are they in the ways they seek to resolve their grievance in terms of the psychological process, the context, the goals, and their behavior?

Intimate partner femicide (where a woman is killed by her partner or ex-partner), is the most common type of homicide of women, accounting for a third to a half of such homicides (Stöckl et al., 2013), and is probably the most common type of targeted and grievance fueled homicide in the world. It is clearly an extreme type of intimate partner violence, it often occurs when the woman has left or has signaled her intention to leave, is not always preceded by violence within the relationship, appears more likely when there has been coercive control within the relationship, is not uncommonly preceded by stalking after the relationship has ended, is associated with previous sexual abuse of the partner and sexual jealousy, and sometimes sexual behavior or abuse may occur proximal to the homicide (Adams, 2016; Matias et al., 2020; Spencer and Stith, 2020). Sexual violence in an intimate relationship may be a marker of coercive control, associated with sexual jealousy, or an indicator of sexual deviance, sexual preoccupation, or sexual attitudes in the perpetrator. Sexual conflict or aggression may be direct precipitants of homicidal violence, so sexual behavior or abuse may occur proximally to the killing, either on the pathway to killing or as part of the homicide (Adams, 2016; Stefanska et al., 2021).

Intimate partner sexual homicides have been found to make up a quarter of sexual homicides in Australia and New Zealand (Eichinger and Darjee, 2021). There has been very little research on intimate partner sexual homicide. Among Stefanska et al.’s (2021) 71 cases in England and Wales there were some apparent commonalties with non-sexual intimate partner homicides (conflict preceding the homicide, occurring in the victim’s home, tending to hand themselves in to police), some commonalties with both intimate partner and sexual homicide findings (intoxication, strangulation) but many findings appear to be very similar to those in non-intimate partner sexual homicides (the types of sexual and non-sexual behaviors inflicted on the victim, the proportion where the sexual behavior was either directly or indirectly linked with the act of killing). They concluded that there were many similarities between intimate partner sexual and non-sexual homicide, and that the indirectly sexual cases involved “punishment” (rage, hatred, jealousy, revenge) whereas the directly sexual cases were often an “act of last possession” including cases where sex occurred post-mortem, which has been termed “pseudonecrophilia” (Meloy, 1996). Preliminary, yet to be published, findings in a cohort from Australia and New Zealand (Eichinger and Darjee, 2021), found that like intimate partner non-sexual homicide, but unlike sexual homicide generally, anger/revenge was the predominant motive. The violent and sexual behaviors were the same as in non-intimate sexual homicides, but intimate partner cases (like non-sexual intimate partner homicide) more often had a precipitant and more often were followed by the offender attempting suicide and/or handing themselves to police. Both samples show that grievance plays a major role in intimate partner sexual homicide, but it remains unclear whether such cases should be viewed primarily as intimate partner homicides or sexual homicides or a unique hybrid.

There has been increasing recognition of and research on intimate partner sexual violence (McOrmond-Plummer et al., 2016a). Perhaps unsurprisingly the motivations for intimate partner sexual violence are like those for intimate partner violence, which as mentioned above overlap with those for non-intimate partner rape. Grievance (including anger, vengeance, and jealousy) is the predominant motivator, which is different from non-intimate partner rape where entitlement/power predominate (Camilleri and Miele, 2016). Although vindictive rapists target strangers, acquaintances and intimate partners, the latter are disproportionately represented among their victims. Indeed, the current authors have observed a large number of vindictive offenses among those who rape their ex-intimate partners. The desire to punish the victim for daring to end the relationship is evident.

As there are usually separate laws, services, risk assessment instruments, management approaches and treatment programs for “sexual offenders” versus “intimate partner” or “family violence offenders,” whether intimate partner sexual violence is viewed as intimate partner violence, sexual offending or both is not just of theoretical interest. Men who rape their partners are often viewed as, and given interventions for, sexual offenders, whereas viewing this type of violence as primarily grievance fueled may more appropriately sit it within approaches, measures and services aimed at intimate partner violence prevention. Unfortunately, little research has been conducted comparing offenders who commit non-intimate partner sexual violence, intimate partner sexual violence and non-sexual intimate partner violence, with one study of a sample in a forensic mental health unit finding that intimate partner sexual violence perpetrators did not closely match either of the other two groups, although they had a few factors in common with each (Camilleri and Quinsey, 2009).

### Honor-based violence and homicide

Honor-based violence is culturally, religiously and/or family sanctioned violence, usually toward a female (daughter, sister, wife, or mother), who is considered to have dishonored and therefore brought shame on the husband, family, and/or wider community group through her actual or perceived sexual behavior (Cooney, 2019). The context is usually one where the female’s sexuality is controlled within a patriarchal and misogynistic culture or group. Honor based violence and homicides are the resolution of the grievance against the girl or woman through her punishment, and are usually committed by fathers, brothers, husbands, or other related males, often acting together. The overlap with precursors to and the context of intimate partner and sexual violence include the socio-culturally sanctioned attitudes toward and expectations of females; leading to male proprietariness, suspiciousness toward females, viewing females as sexually provocative, male entitlement, acceptance of use of control and force; culminating in what is considered by the perpetrator(s), family, and community as virtuous violence. Honor based killings are often symbolically brutal and several cases have involved the rape of the victim.

Although the social context and the process of such violence may be considered different, the parallels with vindictive rape are striking, in terms of motivation (morally rightful punishment), the behavior inflicted (humiliating and vengeful punishment which is brutal and may involve torture and sexual violence), and the attitudes and emotional responses of perpetrators which precede offenses. Also, the attitudes which underpin honor-based violence are very familiar to researchers and practitioners who work with men who perpetrate rape more generally. Indeed, Roberts (2014) has argued that a psychologically orientated motivational model accounts more readily than gender-exclusive or culturally based explanations for the perpetration of violence justified by claims of honor.

### Incels

Incels (involuntary celibates) are an online community of males, defining themselves as unable to have sexual or romantic relationships despite desiring them, and who have an “antiwomen ideology” (Van Brunt and Taylor, 2020). They believe certain biologically pre-determined factors make males attractive to females, these factors are missing or sub-standard in themselves, therefore they are undesirable, rejected and remain virgins. They blame and resent the attractive females and sexually “successful” males. Incels become isolated, lonely, jealous, frustrated, and feel marginalized, with their identities as incels and attitudes to women, the world and themselves perpetuated by their involvement with online fora and communities. Several acts of mass homicide have been committed by self-professed incels, targeting the resented females, males and society more generally, with the perpetrators of these crimes becoming idolized and martyrized by the online incel community (Hoffman et al., 2020). Interestingly there are no descriptions of direct sexual violence committed by incels, although in research they have been found to endorse fantasies of rape, they support the rape of the unattainable females, and they may encourage others to target females in this way (Scaptura and Boyle, 2020). Stickel (2020) found that sexual frustration significantly predicted sexual objectification of women, hostility toward women and acceptance of modern myths about sexual aggression. So, the attitudes of incels toward females overlap with attitudes seen in rapists, including seeing women as sex objects, adversarial beliefs about heterosexual relations, women being deceitful, women being unknowable and entitlement.

They appear to have characteristics in common with males who commit a type of stalking sometimes labeled “incompetent suitors” (Mullen et al., 2009), who are desperate to date an attractive female but lack the interpersonal skills and confidence to go about this appropriately and/or are oblivious to, or unconcerned about, how they are seen by the females they pursue. They also appear to have characteristics in common with the aforementioned *non-sadistic sexualization* group of rapists who have also been labeled “inadequate,” “compensatory” or “power-reassurance.” These men have low-self-esteem, feel powerless and inadequate, are unsure of their manhood, feel sexually incompetent, fantasize about attractive females and a consenting sexual encounter, and act in a “gentlemanly” or “pseudo unselfish” manner (Hazelwood, 1987, 2017) manner, hoping to please the victim sexually. They commit their rapes to compensate for their feelings of inadequacy, by having power within a sexual encounter, and to have sexual intimacy with a female. In the abovementioned victim role model (Canter, 1994; Canter and Youngs, 2012) such offenders assign the role of *person* to their victims and their offenses are behaviorally akin to abusive variants of consensual sexual interactions. Indeed, the underlying fantasy is that the victim will submit and be so pleased by the offender’s sexual prowess that they will invite him to continue and repeat the sexual activity (Cohen et al., 1969). Although incels may harass and stalk females, have fantasies of rape and support the rape of attractive women, there are no reported cases of incels committing sexual violence directly. Rather in a rare minority their resentment, isolation, jealousy, and sense of grievance may, in the context of the well-known markers of hopelessness, last resort thinking and suicidal ideation, fuel vengeful violence toward the unobtainable females, envied males and excluding society. In essence, they transition from sexually fantasizing about hypothetical females assigned to the person role, to non-sexualized violence against females assigned to the vehicle role.

In terms of psychological characteristics, incels have been described as having problems with areas such as negative body image, shyness, anxiety, social skills deficits, autism, sexual and romantic inexperience, loneliness, depression, suicidal ideation and being “off time” relative to their peer’s developing sexuality (Broyd et al., 2022; Moskalenko et al., 2022). However, it is unclear if such issues pre- or post-date self-identification as an incel. It has been reported that “incel traits” (e.g., rejected, insecure, fearful, excluded, shunned, unattractive, hateful, resentful, vengeful) identified in a survey of heterosexual males, were associated with grievances about gender roles and hostile attitudes to women, and with violent fantasies of rape and using powerful weapons against enemies (Scaptura and Boyle, 2020).

So, there are various parallels between the grievances and characteristics of incels and men who commit rape, but although their beliefs and community have been described as an example of “rape culture,” there are few if any examples of incels who have committed direct sexual violence. Perhaps this is because they desire a romantic sexual relationship, they have little contact with women, or they are psychologically and/or physically unable to commit a coercive violent sexual act. It is also important to note that, like other forms of GFV, although many incels endorse the incel ideology, few are “radicalized,” and fewer still enact their grievance through violence (Hoffman et al., 2020).

### Same, similar, or different?

Considering these behaviors as the same or within the same overarching paradigm already occurs within the concept of gender-based violence (Russo and Pirlott, 2006), and policies to reduce and prevent men’s violence toward women (and children; Council of Australian Governments, 2010). This perspective helpfully focuses on the common sociocultural structures and attitudes that underpin these forms of violence, the ways boys and men develop and function in relation to females (including gender roles, intimacy, and sexuality), the role of “toxic masculinity” and “rape culture,” and the socio-political changes needed to address these. However, even in countries with higher levels of gender equality and equity, intimate partner and sexual violence rates may be high (Gracia et al., 2019; Wemrell et al., 2020), and male perceptions of women’s lack of subservience or increased power may fuel the grievances underpinning these forms of violence. In addition, viewing these behaviors as simply manifestations of patriarchy and toxic masculinity does not account for why some men within the same culture and with similar grievances against women enact them in different ways, with some progressing to severe and sometimes fatal forms of sexual and non-sexual violence. A GFV framework which encompasses both sexual and non-sexual violence toward women better allows the consideration of what is the same about such behaviors and perpetrators, what is similar, but also what is different.

## Grievance and sexual violence beyond male rape of females

Having considered the overlaps between GFV toward women and sexual violence we now turn to other forms of sexual violence. The concept of GFV does not appear to apply well to sexual offenses committed against children. In terms of proximal motivation, factors subsumed by grievance, such as anger, vengeance, and jealousy, are rare in sexual offenses against pre-pubertal children, with the proximal drivers being sexual attraction to children, lack of adult intimacy, disinhibition and/or opportunity (Lanning, 1992). Although many men who commit child sexual offenses could be considered to have suffered a grievance, in terms of sexual or other abuse in childhood (Jespersen et al., 2009), a state of feeling aggrieved focused on children is uncommon, and their offenses are not usually enacted to resolve a grievance. There are cases where men sexually abuse children of partners or ex-partners to enact a grievance against the woman, men who commit intimate partner violence may be more likely to abuse their children including sexually (Bidarra et al., 2016) and much child sexual abuse is clearly targeted, but in our view, it would be a stretch to fit the vast majority of child sexual abuse within the GFV paradigm. Indeed, this is perhaps one of the biggest differences between males who sexually victimize adult women and those that sexually victimize children. When offenders commit violent or homicidal sexual offenses against children, unlike with adult victims, grievance (or anger) rarely plays a role (Chopin and Beauregard, 2019), although sexual sadism sometimes does (Lanning, 2010; Chopin and Beauregard, 2022).

Although most sexual violence is committed by men against women and children, there has been increasing recognition of sexual offending perpetrated by females (Gannon and Cortoni, 2010). The largest group of females who commit sexual offenses are those who act alongside, and often under the influence of, a male (Harris, 2010; Cortoni and Gannon, 2016). Most other sexual offenses committed by females are against their children or adolescents. The smallest group commit offenses against adults (Harris, 2010), they victimize women more often than men, but sexual violence by women against men is rarely reported (Depraetere et al., 2020). When women perpetrate sexual offenses against men, grievance toward the man or men in general is often the driving factor. This is seen in very rare cases of homicides with a sexual element committed by females either on their own or while leading others (Skott et al., 2019; Clarkson et al., 2020). Getting revenge on men by luring them with sex and then killing them is a pattern seen in some serial homicides committed by women (Arrigo and Griffin, 2004; Pettigrew, 2019).

There has also been recognition of male victimization by other males, however although there have been studies of the impact, legal and sociocultural context of this type of sexual violence (Lowe and Rogers, 2017), there have been few studies of its perpetrators. Groth and Burgess (1980) stated that these were “acts of retaliation, an expression of power, and an assertion of their strength and manhood.” In a study of male-on-male sexual homicide the most common of three types was the “avenger.” This type of offender was “avenging himself directly on his partner for all the grievances (present or past) that he feels he has been a victim of” (Beauregard and Proulx, 2007). More recently, Lundrigan and Mueller-Johnson (2013) have posited a typology of male stranger rape based on the victim-offender interaction. Through multivariate analysis they identified three such themes of interaction: *hostility*, *involvement intimacy*, and *involvement exploitation*. Interestingly, these align quite closely with the victim role model described above (vehicle, person, and object respectively). Using a strict criterion for assigning individual cases to themes, Lundrigan and Mueller-Johnson found that 80 percent could be assigned to a dominant theme, with 17 percent primarily reflecting hostility. This was lower than some previous research that suggested that in more than half of male rape cases force and physical injury was described (e.g., Walker et al., 2005). Moreover, a subsequent study by Lundrigan (2014) indicated that male victims were more likely than female victims to experience overtly hostile interactions. In addition, Ioannou et al. (2017) argued that “most male-on-male sexual assaults are violent in nature” (p. 189). Accordingly, a large number of male victim sexual offenses arguably involve considerable grievance as part of the offender’s motivation. However, it is unclear if these are offenders that are solely high on the grievance continuum, or whether they are also deviant and located high on the agonistic continuum as well.

The role of grievance in the most extreme form of sexual violence, sexual homicide, was highlighted by the systematic review of typologies by Higgs et al. (2017a). This identified three types: sexualized murder driven by sexual deviance, often sexual sadism; rape murder, committed instrumentally or reactively in an attempt to avoid detection; and grievance murder (driven by anger, vengeance, rage or hatred). These overlap with typologies of rapists and rape (see above), although the “power-reassurance,” compensatory or inadequate type of rapist (non-sadistic sexualization type) does not have a sexually homicidal analog. As highlighted above, grievance is relevant to sexual homicides committed by men against women, by men against men, by men against intimate partners, and by women against men. These are the extreme and fatal equivalents of vindictive and angry rape and would seem to fit squarely within the paradigm of GFV. But like with rapists, if grievance is conceptualized more broadly to encompass a wider range of attitudes toward women and ways of enacting, reacting to or compensating for them, grievance can be viewed as a relevant issue in most sexual homicides.

## Implications

### Research

We have set out the case for why at least some forms of sexual violence should come within the scope of GFV, drawing together theory, research and case examples. We now need to better understand the components of grievance across different kinds of GVF to understand how this develops, what triggers it, how individuals do or do not cope with it and under what circumstances it leads to violence. Consideration of the development of grievances and how they play out in an individual’s life need to include understanding of biological, psychological, and social factors. How are grievances triggered and are there factors that explain why this leads to planned or actual violence? This understanding could lead to the development of a typology of GFV applicable to a spectrum of offending. Our growing understanding of childhood adversity and how it can impact on the development of the brain could inform both knowledge about the development of grievances that precede GVF as well as how best to work with people who have carried out this behavior. Qualitative research based upon interviews of individuals who have committed violence that includes consideration of trauma and abuse to understand how grievance has developed and played out across childhood, adolescence and adulthood could provide valuable insights. Life Maps can be used in assessment to understand an individual’s patterns of thinking as well as providing information about trauma and adversity to increase understand of psychological functioning across their lifetime as well as that related to an individual’s actual offending. While Life Maps can be used for working therapeutically with an individual (Carter and Perkins, 2018), we propose this sort of approach could be adopted to develop our understanding of Grievance empirically.

In addition, if grievance does indeed fuel violence, the development of risk assessment protocols will benefit from a clearer empirical picture of its valid measurement, risk relevance of measured change, and how it interacts with other key risk factors. We know that risk increases cumulatively with the number of static and dynamic risk factors that are identified, but there may be advantages to examining the potential impact of one dynamic factor on another. Could targeting grievance indirectly act upon associated risk factors? For example, if risk associated with sexual deviance were to be tempered through its relationship with grievance in individuals who have used sexual violence, both risk assessment and intervention would benefit from further research on the processes involved.

It is not unusual for people in general to hold grievances, be it temporarily in a certain situation related to a specific set of circumstances. There are of course individuals who may hold grievances but never offend. For example, someone who has suffered medical negligence, could hold a grievance toward a hospital trust where they suffered this negligence. Some level of grievance in this situation could be understandable. At the same time, there could become a point or situation whereby that grievance starts to impact negatively on a person’s life, driving unhelpful or even problematic behavior. Given that grievance is not limited to those who commit violence, the starting point for better a better understanding GVF that can inform prevention of offending and how to ameliorate risk with those that do offend is to validate that grievance sits on a continuum. The extent to which grievances develop, their intensity, duration, how they are triggered and what they might lead to whether this is criminal or resolved in a pro-social way is likely to be an interplay of individual biological and psychological factors with social factors. Research needs to explore and develop this understanding.

### Treatment

The very presence of grievances and the patterns of thinking or schema that can trigger and fuel them can be an obstacle to the development of therapeutic alliance, which is necessary for an individual to be able to change attitudes related to offending and develop skills to more effectively self-regulate and solve problems in a pro-social way. Addressing grievance thinking early in any rehabilitative efforts may help the individual concerned to be able to engage fully in treatment and make progress. Another therapeutic advantage is afforded by the accessibility of grievance as a concept. This is something that may be quite readily understood from both sides of the therapeutic relationship, and it lends itself to collaborative working. It provides a framework suited to privileging accessible language and bringing about change through gaining an understanding of the function of grievance thinking and behavioral responses as well as triggers. Targeting grievance may prevent any sort of GVF, especially if the individual is able to identify and consider antecedents where they can take appropriate action or enlist support. Importantly, identifying and addressing grievance when individuals first come into correctional settings could reduce the risk of their going on to commit more serious GFV.

## Conclusion

The similarities between grievance-fueled nonsexual violence and sexual violence are such that the GFV paradigm appears to apply well to working with men who have used sexual violence. Furthermore, people who have committed sexual offenses tend to have also committed nonsexual offenses, and the possibility of the inverse cannot be excluded: Sexual elements are apparent in some acts of GFV, and trajectories toward sexual violence compared to those seen in GFV overlap in ways that suggest that certain individuals inclined toward GFV might be susceptible to using sexual violence depending on situational factors such as socio-political context and interpersonal experiences, and in turn, psychosocial development where that leans toward movement along the agonistic continuum. Thus, identifying and tackling grievance as early as possible is likely to be beneficial for many individuals.

We have proposed a dimensional conceptualization of grievance, which captures different forms of grievance and suggests that these can be present to different degrees of severity. We additionally posit that they fuel violence depending on the interaction of grievance with other factors that likely influence both the trajectory from threat to action and the route to either sexual or nonsexual violence. The agonistic continuum represents one such component to offending behavior that we hypothesize to play a critical role in grievance-fueled sexual violence. As well as a need to test the model, other components that underlie certain types of sexual and nonsexual violence will need to be examined. For example, it will be necessary to consider how the violent offending of individuals with strong psychopathic traits fits (or not) in a grievance-agonistic conceptualization. However, we believe that the parallels that we have drawn between the sexual violence literature and the GFV paradigm point to a new direction for future research with significant potential for informing threat assessment and correctional policies.

## Data availability statement

The original contributions presented in the study are included in the article/supplementary material, further inquiries can be directed to the corresponding author.

## Author contributions

All authors listed have made a substantial, direct, and intellectual contribution to the work and approved it for publication.

## Conflict of interest

The authors declare that the research was conducted in the absence of any commercial or financial relationships that could be construed as a potential conflict of interest.

## Publisher’s note

All claims expressed in this article are solely those of the authors and do not necessarily represent those of their affiliated organizations, or those of the publisher, the editors and the reviewers. Any product that may be evaluated in this article, or claim that may be made by its manufacturer, is not guaranteed or endorsed by the publisher.

## Footnotes

^1^In total there are nine rapist types in the MTC:R3 system and eight in the MTC:R4, based on the four motivations of opportunity, pervasive anger, sexual gratification, and vindictiveness. The remaining types are based on ratings of high or low social competence for the opportunistic, non-sadistic sexualization, and vindictive types (see Knight and Prentky, 1990; Knight, 1999).

^2^The muted sadist was included in the MTC:R3 typology, but was removed from the MTC:R4 revision, largely because of reliability issues (i.e., their offences appear behaviorally similar to the non-sadistic sexualization types, but they also share sadistic fantasies with the overt sadistic types; see Knight, 2010).
